# Engineering of *Bacillus thuringiensis* Cry Proteins to Enhance the Activity against Western Corn Rootworm

**DOI:** 10.3390/toxins11030162

**Published:** 2019-03-14

**Authors:** Jingtong Hou, Ruth Cong, Michi Izumi-Willcoxon, Hana Ali, Yi Zheng, Ericka Bermudez, Mark McDonald, Mark Nelson, Takashi Yamamoto

**Affiliations:** 1 Corteva Agrisciences, Agriculture Division of DowDuPont, Hayward, CA 94545, USA; Rcong93@yahoo.com (R.C.); michi@secondgenome.com (M.I.-W.); hnaa.ali@gmail.com (H.A.); zyi68@hotmail.com (Y.Z.); erbgmf@gmail.com (E.B.); p.gibson1@comcast.net (M.M.); 2 Corteva Agrisciences, Agriculture Division of DowDuPont, Johnston, IA 50131, USA; mark.e.nelson@corteva.com

**Keywords:** *Bacillus thuringiensis*, Novel Cry protein, Maltose binding protein, DNA shuffling, Western corn rootworm

## Abstract

A novel *Bacillus thuringiensis* Cry protein, Cry8Hb, active against *Diabrotica virgifera virgifera* (Western corn rootworm, WCRW) was discovered. Unexpectedly, the anti-rootworm activity of the Cry8Hb toxin was enhanced significantly by fusing *Escherichia coli* maltose binding protein (MBP) to this Cry toxin. While the exact mechanism of the activity enhancement remains indefinite, it is probable that the enhancement is a result of increased solubility of the MBP-Cry8Hb fusion in the rootworm midgut. This hypothesis was examined using a synthetic Cry3 protein called IP3-1, which was not soluble at a neutral pH like Cry8Hb and marginally active to WCRW. When IP3-1 was fused to MBP, its anti-WCRW activity was enhanced 13-fold. To further test the hypothesis, DNA shuffling was performed on IP3-1 to increase the solubility without MBP. Screening of shuffled libraries found six new IP3 variants showing very high anti-WCRW activity without MBP. Sequence and 3D structure analysis of those highly active, shuffled IP3 variants revealed several charge-altering mutations such as Lys to Glu on the putative MBP-attaching side of the IP3 molecule. It is likely that those mutations make the protein acidic to substitute the functions of MBP including enhancing the solubility of IP3 at a neutral pH.

## 1. Introduction

*Bacillus thuringiensis* (Bt), a spore-forming bacterium, is known for its pathogenicity to insects including agricultural pests. When Bt sporulates, it produces crystalline inclusion bodies that contain one or more proteins called Cry proteins. Some of the Cry proteins are highly active against certain insect species, for example Cry1Aa against silkworm. While many Cry proteins are active against lepidopteran insects, only a few are known to be active against *Diabrotica* species. Cry34Ab and Cry35Ab [1] and modified Cry3 proteins such as Cry3Bb [2] have been utilized in transgenic corn to control the *Diabrotica* complex, particularly *Diabrotica virgifera virgifera* (Western corn rootworm, WCRW). The wild-type Cry3 proteins are known for their high activity against coleopteran species, for example, *Leptinotarsa decemlineata* (Colorado potato beetle), but their activity against corn rootworm, particularly WCRW, is not high enough for commercial application in transgenic corn. Walters et al. [3] found that mutations enhanced the anti-WCRW activity of Cry3Aa. They inserted a cathepsin recognition sequence in the loop between α-helices 3 and 4 in Domain I. Cathepsins that belong to the cysteine protease family have been identified as the major proteases found in the corn rootworm digestive system [4]. The other Cry3 protein, Cry3Bb, has been found to be active against WCRW [5]. Vaughn et al. [2] engineered Cry3Bb to enhance its activity against WCRW. Both modified Cry3Aa and Cry3Bb proteins have been shown to be effective in transgenic Bt-corn to control the rootworm complex.

Other WCRW-active Cry proteins include Cry8Bb [6] and Cry8Hb (this study). We discovered that the anti-rootworm activity of Cry8 proteins was significantly enhanced when they were fused to *Escherichia coli* maltose binding protein (MBP). A similar observation was made with a synthetic Cry3 called IP3-1. The activity enhancement of IP3-1 with MBP was extraordinarily high. It appears MBP increases the solubility of the WCRW-active Cry proteins in a neutral pH solution similar to WCRW gut digestive juice and enhances the insecticidal activity. Therefore, we applied the DNA shuffling technology described by Stemmer [7,8] to elucidate the functions of MBP. DNA shuffling is a powerful tool to generate highly diversified sequences artificially. The shuffled library was screened for anti-WCRW activity, and the relationship between sequence and activity was examined.

During this study, a high throughput screening method was developed for WCRW based on an existing method designed for lepidopteran insect species [9].

## 2. Results

### 2.1. WCRW-Active Bt Isolate and Its Cry Protein

**Figure 1:**
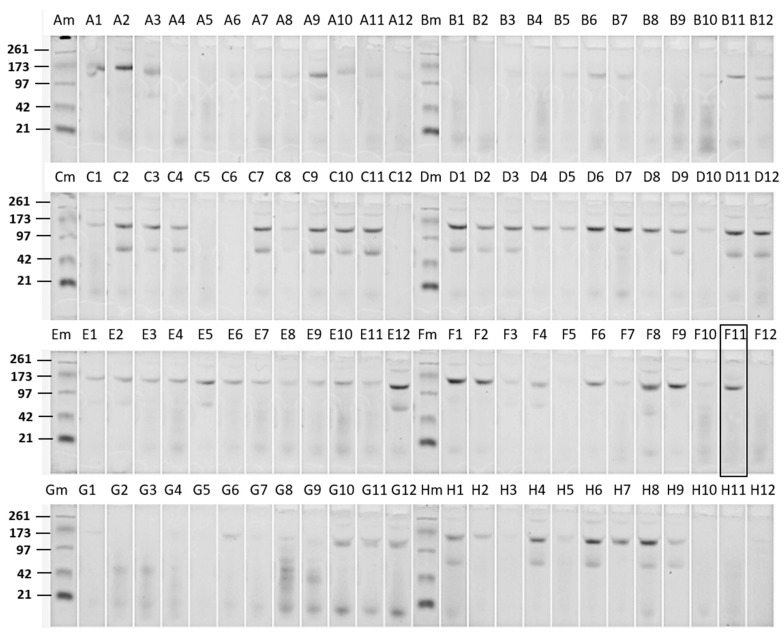
An example of SDS-PAGE analysis of Cry proteins isolated from Bt strains arrayed in one seed culture plate, DP7. In this plate of screening 96 Bt strains, one strain, F11 (boxed), was found to be active against WCRW. For the analysis, E-PAGE™ Precast Gel System from Life Technologies was used. E-PAGE has 96 sample lanes and eight marker lanes (Am–Hm), in which, E-PAGE™ SeeBlue^®^ Protein Standard was loaded. The molecular weights of the standards are shown on Row A, C, E, and G. The gel was stained with Coomassie Blue. Digital image of the stained gel was aligned by rows utilizing Life Technologies’ E-Editor™ Software.

**Figure 2:**
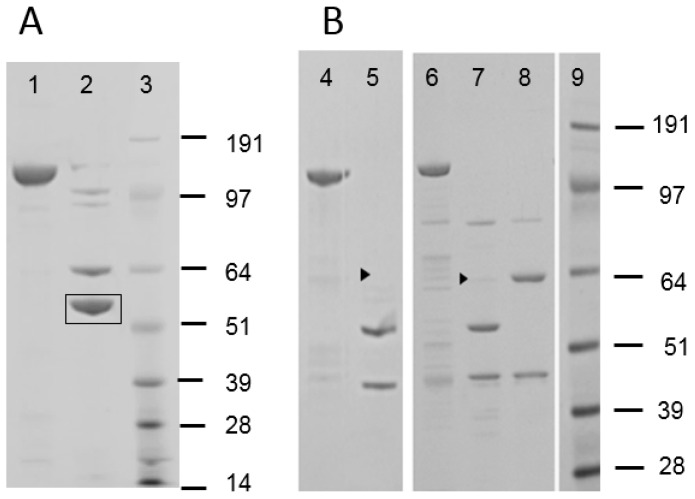
SDS-PAGE analysis of the Cry8Hb protein (protoxin) isolated from DP7-F11 (Panel **A**) and MBP fusions of Cry8Hb and IP3 toxins (toxin part only) (Panel **B**). Panel **A**, Lane 1, 135 kDa Cry8Hb from DP7-F11; Lane 2, trypsin-digested 65 kDa and 55 kDa fragments of the DP7-F11 protein. Panel **B**, Lane 4, MBP-Cry8Hb(toxin); Lane 5, trypsin-digested MBP-Cry8Hb; Lane 6, MBP-IP3-1; Lane 7, trypsin-digested MPB-IP3-1; Lane 8, trypsin-digested MBP-IP3-7. Very faint bands at 65 kDa (arrowheads) were visible in Lane 5 (Cry8Hb) and Lane 7 (IP3-1) but not reproduced clearly in the photograph. Lanes 3 and 9 were SeeBlue^®^ Plus-2 protein standard with approximate molecular weights in kDa. The stained SDS-PAGE gel, Panel **A**, Lane 2, was blotted on a sheet of PVDF membrane, and the 55 kDa band (boxed) was excised for N-terminal sequencing at Stanford University Protein and Nucleic Acid Facility.

Bt crystal protein samples isolated from a large number of naturally occurring Bt strains were screened against WCRW. Figure 1 shows an E-PAGE image of one plate-full of Cry proteins. This plate contained approx. 60% of Bt isolates showing typical 130 kDa Cry proteins, and some of those had additional 70 kDa proteins which could be truncated Cry proteins such as Cry2 and Cry3. All other plates showed patterns similar to this plate. Only one sample from a particular Bt strain, DP7-F11, showed significant activity against WCRW. As shown in Figure 1, the crystal protein preparation from DP7-F11 produced one band at about 130 kDa. A large amount of the Cry protein was prepared from flask-grown DP7-F11 (Figure 2, Panel A, Lane 1) and subjected to further characterizations. When the Cry protein was digested with trypsin, two major polypeptides, one around 65 kDa and the other about 55 kDa, were observed (Figure 2, Lane 2). The trypsin digestion pattern suggested that the 130 kDa protein is a protoxin like WCRW-active Cry8Bb whose mature toxin has a protease-sensitive loop between α-helices 3 and 4 [10]. The N-terminal sequences of the DP7-F11 55 kDa protein extracted from the SDS-PAGE gel were determined by Edman degradation method as SVTNIRSQFETVNNFF without any ambiguity at each step. The high-quality sequence suggests that the 55 kDa band contains only one protein. A BLAST search of this Edman sequence against known Bt Cry proteins showed significant homology to Cry8Ha, matching 11 residues out of 16, and Cry7Ja, matching 9 out of 16.

As described in Materials and Methods, a novel *cry* gene tentatively called RX002 was cloned, and its complete nucleotide sequence encoding a 133 kDa Cry protoxin was determined. The nucleotide sequence was submitted to the NCBI Genbank, and an accession number, KP713881, was assigned. The N-terminal sequence of the 55-kDa trypsin-digested polypeptide starts at Ser165, directly following Arg164, a possible trypsin site. No attempt was made to determine the C-terminus of the 55 kDa protein, but it was presumed to be Lys669 or Lys670 based on the size and previous reports of trypsin activation of Cry1A-type proteins [11]. The calculated molecular weight of the theoretical polypeptide of Ser165-Lys669 is 56.5 kDa. No N-terminal sequencing was done with the 65 kDa polypeptide. However, based on the size determined by SDS-PAGE and alignments with other well-characterized Cry proteins, the N-terminal amino acid for the 65 kDa polypeptide is likely to be Ala75. Trypsin could digest Lys74 to produce a polypeptide from Ala75 to Lys699 whose calculated molecular weight is 66 kDa.

A BLAST search of the whole RX002 peptide sequence confirmed that the closest homologue was Cry8Ha, as predicted from the N-terminal sequence of the 55 kDa trypsin-digested polypeptide. It is expected that RX002 and Cry8Ha are three domain-type Bt Cry proteins structurally similar to Cry3Aa whose 3D structure has been determined by X-ray crystallography [12]. Domain assignments of RX002 and Cry8Ha were made by aligning those sequences with that of Cry3Aa. The alignment showed that RX002 and Cry8Ha have almost identical Domain I and III, but Domain II and the protoxin region sequences were somewhat different. Therefore, the *Bacillus thuringiensis* Toxin Nomenclature Committee [13] assigned a new holotype Cry name, Cry8Hb1, to RX002.

The gene encoding Cry8Hb was cloned in pMAXY3206 and expressed in a plasmid-cured, *cry*-minus Bt host called G8 [9]. The recombinant Bt produced inclusion bodies which were dissolved easily in 2% 2-mercaptoethanol with NaOH added to make it pH10.5. The protoxin was purified by size-exclusion column chromatography and exposed to trypsin. SDS-PAGE analysis of trypsin-digested Cry8Hb produced a 65 kDa polypeptide along with the 55 kDa fragment. The digestion pattern was identical to the pattern observed with the Cry protein prepared from DP7-F11. The Cry8Hb protoxin purified from the recombinant Bt was active against WCRW. EC_50_ of the protoxin protein was estimated to be approximately 700 ppm, which is equivalent to 350 ppm by toxin weight.

### 2.2. MBP Enhances the Anti-WCRW Activity of Cry8Hb

A 5′ portion of the *cry8Hb* gene encoding a 75 kDa polypeptide (Met1- Lys699) was cloned in pVER6805 vector and expressed as a fusion with MBP. The fusion protein was purified by Ni-NTA affinity chromatography utilizing the His-tag attached to MBP (Figure 2, Panel B, Lane 4). Trypsin digestion of MBP-Cry8Hb produced a minor 65 kDa and a major 55 kDa polypeptides (Figure 2, Lane 5). The 65 kDa band in the SDS-PAGE gel was very faint, indicating that the digestion at the α3-4 loop was highly efficient when fused with MBP. There was a 44 kDa polypeptide band. The size of this band indicates the polypeptide is MBP whose theoretical molecular weight in pVER6805 is 43.8 kDa. Only the 44 kDa was positive with mouse anti-MBP antibody by Western blotting. Judging from the size of MBP produced by the trypsin digestion, it appeared that the protease-cleaved Arg in the Factor Xa recognition sequence (IEGR) engineered at the end of MBP in the original pMAL. In addition, it is possible that trypsin digests at the second Arg residue of Cry8Hb. However, the second Arg residue is not likely to be a site for trypsin, as this Arg residue is followed by Pro. The junction amino acid sequence between MBP and Cry8Hb was as follows.

… (MBP)…IEG**R**ISELG–M**R**PNN…(Cry8Hb)...

When both MBP-Cry8Hb and the trypsin-digested sample were assayed against WCRW using *N* = 3 (18 wells per dose of each sample), it was found that the MBP fusion was more active. EC_50_ of MBP-Cry8Hb was 178 ppm with 145–220 ppm 95% confidence limits while EC_50_ of the trypsin digest was 390 ppm with 325–483 ppm 95% confidence limits. For comparison, both EC_50_ values were calculated based on the weight of the 65-kDa portion, which is the active part of Cry8Hb without MBP after the concentrations of the MBP-fusion and non-fusion were determined by SDS-PAGE. There was no significant difference in the anti-WCRW activity of the trypsin-digested Cry8Hb between two samples prepared from Bt as a protoxin and *E. coli* as an MBP fusion.

The MBP-Cry8Hb fusion was highly soluble in the HEPES buffer, but its trypsin-digested Cry8Hb was not. The pH of the digested sample was raised to pH10.5, and Superdex 200 column chromatography was performed with 25 mM CAPS-NaOH buffer, pH10 to purify the trypsin-digested Cry8Hb away from MBP. Similarly, the Bt-made Cry8Hb protoxin was highly soluble in a neutral pH buffer such as Tris-HCl buffer, pH8 but precipitated within a few minutes during the trypsin digestion.

### 2.3. DNA Shuffling of Cry3-Type Protein

The DNA shuffling technology described by Stemmer [7,8] was applied to examine the relationship between solubility and activity of a Cry3 protein. This was to test the hypothesis that increasing the solubility of the Cry toxin in the environment of WCRW midgut enhances the activity, at least in a similar pH range. A new Cry3-type gene, termed IP3-1, was synthesized and used as the parent gene for DNA shuffling. The IP3-1 peptide sequence was produced by computer-assisted protein design using the Cry3-family proteins. IP3-1 peptide sequence differed from that of Cry3Aa at the following residues: W106L (the 106th amino acid residue, Trp, in Cry3Aa was replaced with Leu), M117I, V140F, I186V, F206L, K230H, S258T, P292S, E294G, F346L, G468A, L491F, M503T, R531G, and I593M. The IP3-1 gene was cloned in pVER6805 and expressed as an MBP fusion with a poly His tag. The 6XHis-MBP-IP3-1 fusion was highly expressed in the cytoplasm of *E. coli* BL21(DE3) cells as a soluble protein. The protein was purified by Ni-NTA affinity chromatography (Figure 2, Panel B, Lane 6) and then digested with trypsin. Within a few minutes of trypsin digestion at pH8, the clear MBP fusion solution became turbid indicating that MBP removal reduced the solubility of IP3-1. SDS-PAGE analysis showed that the digestion produced a minor 65 kDa polypeptide along with a major 55-kDa polypeptide in addition to a 44-kDa polypeptide (Figure 2, Lane 7) like Cry8Hb. The 55 kDa polypeptide suggested that trypsin cleaved the loop between α-helices 3 and 4. Western blotting using anti-MBP antibody confirmed that the 44 kDa polypeptide was MBP. Since this IP3-1 parent protein was not soluble without MBP in the HEPES buffer at pH8, it was further mutated by DNA shuffling.

After shuffled IP3 DNA was cloned in *E. coli*, several clones were picked at random and sequenced before the library was screened for intended traits. Sequencing revealed that the picked clones differ from the parent IP3-1 by 5 to 10 amino acid mutations. The desired traits were not only high solubility, but also anti-WCRW activity without MBP. However, it is possible that shuffling would produce variants with high solubility but no or little anti-WCRW activity due to one or more detrimental mutation(s). Therefore, the shuffled library was screened directly by anti-WCRW activity after the fusion proteins were digested with trypsin to detach MBP, then the solubility of those active proteins was examined. Since no anti-WCRW activity was observed with purified MBP, no further purification was made after the digestion for high throughput screening that was necessary after DNA shuffling. The protein concentration of the assay samples was set at 0.1 mg/mL, which was 17 ppm when mixed in the diet. The screening identified six clones showing at least 50% insect responses. Those six proteins were numbered IP3-2 to IP3-7.

MBP-fusion proteins of these highly active, shuffled IP3 variants were produced in flasks and purified by large-scale Ni-NTA affinity chromatography. Concentrations of the purified proteins were set at 2 mg/mL in 50 mM HEPES-NaOH buffer, pH8 and then digested with trypsin. None of those highly active IP3 variants precipitated during the digestion, indicating increased solubility by DNA shuffling at least up to 2 mg/mL. After 1-h digestion, the buffer pH in an aliquot was lowered to pH7 with a small volume of HCl to confirm the solubility at pH7. No precipitation was observed with any of those shuffled IP3 variants at pH7. Trypsin digestion of the MBP fusions of those shuffled IP3 variants was analyzed by SDS-PAGE. Figure 2, Lane 8 shows an example with MBP-IP3-7. SDS-PAGE revealed that MBP-IP3-7 was digested with trypsin into two 65 and 44 kDa polypeptides. Unlike the cases of MBP-IP3-1 (Figure 2, Lane 7), no 55 kDa band was observed with MBP-IP3-7 (Lane 8). All other shuffled, highly active IP3 variants had SDS-PAGE profiles similar to that of IP3-7 indicating that trypsin failed to digest the loop between α-helices 3 and 4.

### 2.4. Size Exclusion Chromatography of Trypsin-Digested IP3 Proteins

**Figure 3:**
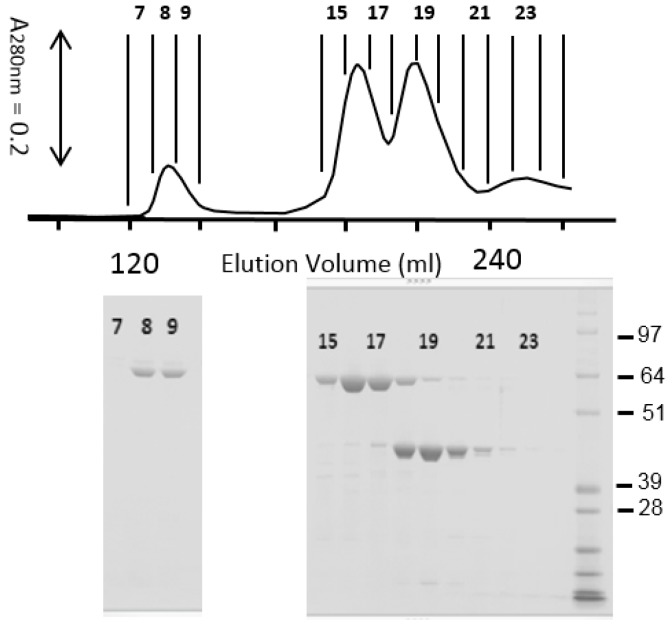
Superdex 200 column chromatography of trypsin-digested MBP-IP3-7 showing most of 65 kDa, MBP-free IP3-7 molecules were monomeric eluting at approx. 195 mL elution volume (Fraction 16) from this GE 26/70 XK preparative column. The 44 kDa MBP appeared at approx. 215 mL elution volume (Fractions 18–19). For this example, 5 mL of 2 mg/mL MBP-P3-7 were digested with trypsin as described in Materials and Methods, and the whole mixture was loaded into the column without any pH adjustment. The elution was made with 25 mM Tris-HCl buffer, pH8 at 2 mL/min using a Gilson 306 HPLC pump under TRILUTION^®^ LC computer software control for high elution volume reproducibility. The column eluate was monitored with a UV detector at 280 nm and collected by fraction collector starting at 72 mL elution volume as shown on the top of the chromatogram. Each fraction was 8 mL. Selected fractions, 7 to 9 and 15 to 24, were analyzed by SDS-PAGE. Numbers on the PAGE gel indicate the fraction numbers corresponding to those on the top of the chromatogram. A protein size marker, SeeBlue^®^ Plus-2, was included in the last (right side) lane with approximate molecular weights in kDa.

Superdex 200 column chromatography was used to separate the trypsin-activated, shuffled IP3 proteins from MBP. Since no precipitation was observed during the trypsin digestion at pH8, the digestion mixture was loaded to the Superdex column without any pH adjustment. The elution was made with 25 mM Tris-HCl buffer, pH8. Figure 3 shows an example of Superdex chromatography conducted with IP3-7. Most of the 65 kDa polypeptide was eluted in Fractions 15–17 (peaked at 195 mL elution volume). At this elution volume, ~70-kDa reference proteins such as bovine serum albumin were expected to show up. Since the 65 kDa polypeptide in Fractions 15–17 was toxic to WCRW, it was considered to be the mature IP3 toxin. This elution volume suggested that the trypsin-digested IP3-7 was monomeric at pH8. All other shuffled IP3 variants selected for high anti-WCRW activity showed very similar chromatography patterns (data not shown) confirming that those shuffled, MBP-free IP3 molecules were monomeric at pH8. SDS-PAGE indicated the peak in Fractions 18–19 contained mostly a polypeptide of 44 kDa. This polypeptide was determined to be MBP with the anti-MBP antibody. No significant anti-WCRW activity comparable to the 65 kDa protein was found in this 44 kDa MBP sample.

**Figure 4:**
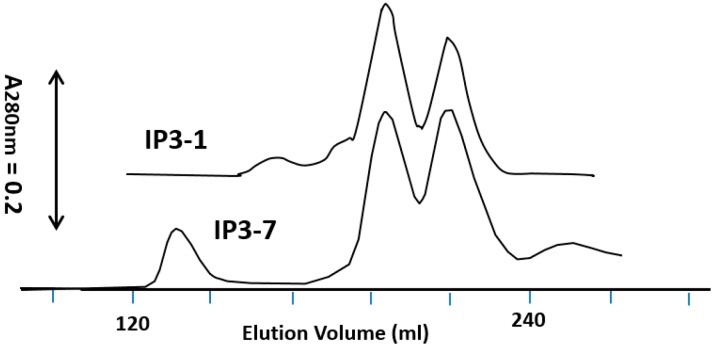
Superdex 200 column chromatography of trypsin-digested MBP-IP3-1 (upper trace) overlaid on the top of the trance of trypsin-digested MBP-IP3-7 (lower trace from Figure 3). After trypsin digestion of IP3-1, the protein was solubilized by increasing pH with 25 mM CAPS and NaOH to pH 10.5 and loaded to the column. Other experimental conditions were identical to those described in Figure 3 except for 25 mM CAPS-NaOH buffer, pH10 used as the elution solvent for IP3-1.

On the other hand, IP3-1 required high-pH CAPS buffer to conduct the chromatography (Figure 4). The IP3-1 protein precipitated during trypsin digestion under the same condition as those of shuffled IP3 in 50 mM Tris-HCl buffer, pH8 indicating insolubility at this pH and concentration. Therefore, pH of the digestion mixture was raised to pH10.5 before it was loaded to the Superdex column. At pH10, the IP3-1 toxin, mostly 55 kDa by SDS-PAGE, was eluted at an elution volume similar to that of shuffled IP3 proteins (Figure 4). The elution volume of the trypsin-digested IP3-1 protein indicated that the size of the protein is around 70 kDa, not 55 kDa as shown by SDS-PAGE. It is likely that the first three alpha helices, 1–3, were still attached to the remaining IP3-1 protein after trypsin cleaved the loop between α-helices 3–4 until it was exposed to SDS.

### 2.5. Activity of Shuffled IP3 Proteins to WCRW

Dose-response assays determined EC_50_ values of those selected IP3 variants. A summary of the assay results is shown in Table 1. All shuffled IP3 variants were highly active without MBP while the parent IP3-1 required MBP to reach the level of the activity similar to those of the shuffled variants. Those six highly active clones were sequenced. DNA shuffling added 6 to 8 mutations to the IP3-1 sequence (Table 2A and Table 2B, gray shade). Among those mutations, several Lys residues were mutated to Glu, possibly making the protein more acidic. When the shuffled IP3 sequences were compared with Cry3Aa, there were 21 to 23 mutations.

### 2.6. Analysis of Mutations Found in IP3 Shuffled Variants

Possible structural modifications arising from mutations occurred to IP3-1 by DNA shuffling were analyzed on the 3D X-ray structure of Cry3Aa [12] and summarized in Table 2B. Amino acid sequence diversities introduced to IP3-1 and its shuffled variants are relatively conserved with the sequence of Cry3Aa. It is likely that the diversities cause no significant changes to the Cry3Aa 3D structure, especially the protein backbone folding. Indeed, a 3D structural model of IP3-1 built with Accelrys Discovery Studio software suite utilizing the homology modeling subroutine MODELER 9.15 [14] showed no significant differences from the Cry3Aa structure. Table 2B lists 2D structure assignments, side-chain solvent exposure, hydrophobicity indices and pKa values of amino acid residues specific to IP3-1 and those mutated by shuffling. At the bottom of the table, average hydrophobicity and pKa of all amino acid residues specific to each IP3 variant are listed. Hydrophobicity indices were adjusted by their corresponding side-chain solvent exposure percentages. For pKa, only those residues, charged atoms of which were fully exposed, were included in the average pKa calculation. In shuffled, activity-enhanced IP3 variants, several charge-converting mutations such as Lys mutated to Glu were found on solvent-exposed residues. The analysis indicated that the mutations introduced by shuffling (gray shaded) made the IP3 variants more acidic than the parent IP3-1 protein. As shown in the bottom row of Table 2B, average pKa values of shuffled IP3 proteins were lower than that of IP3-1 by 0.7 to 2.7. On the other hand, no significant changes in hydrophobicity were induced by shuffling. Interestingly, IP3-7, which showed the highest anti-WCRW activity, had the lowest average pKa.

**Figure 5:**
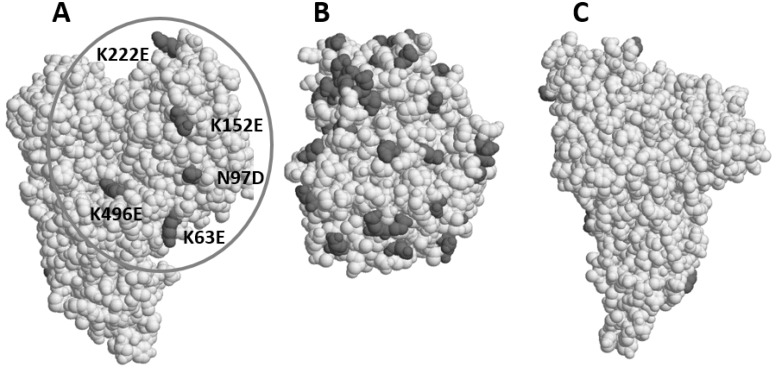
The 3D X-ray structure of Cry3Aa (Panel **A** and **C**) on which pKa-reducing mutations introduced by DNA shuffling are mapped and shown in dark gray. Those mutations are clustered on one side (**A**) of the protein on which MBP is believed to occupy when fused to IP3. In Panel **B**, negatively charged Asp and Glu residues on MBP are shown in dark gray on the solvent-exposed side, which is presumably not attaching to IP3 or Cry3Aa. Panel **C** shows a view of the other side of Cry3Aa, opposite to Panel A. No mutations are visible on this side displayed in Panel **C**.

Mutations introduced by DNA shuffling were mapped on the Cry3Aa structure (Figure 5). It was found that most pKa−reducing (acidifying) mutations, such as Lys to Glu, were clustered on one side of the IP3 protein molecule (Figure 5, Panel A). There was no such mutation on the opposite side (Panel C). Since the coordinates of the first 60 amino acid residues are not available in the Cry3Aa X-ray structure, the orientation of MBP fused to the N-terminus of IP3 remains uncertain. However, it seems that MBP is on the side of IP3-1 shown in Panel A. This assumption is based on a deduced structure of this part of the protein from the Cry2Aa structure [15]. Those five mutations found in the surface area (Panel A, circled) which is presumably covered by MBP (Panel B) may assume MBP’s solubility enhancement role. Indeed, MBP has quite a few acidic or negatively charged, amino acid residues on its surface (Panel B). This suggests that the negative charges on a particular side of IP3 molecule as shown in Panel A is important for the high anti-WCRW activity.

### 2.7. Processing of MBP-IP3-7 in WCRW Gut Fluid 

**Figure 6:**
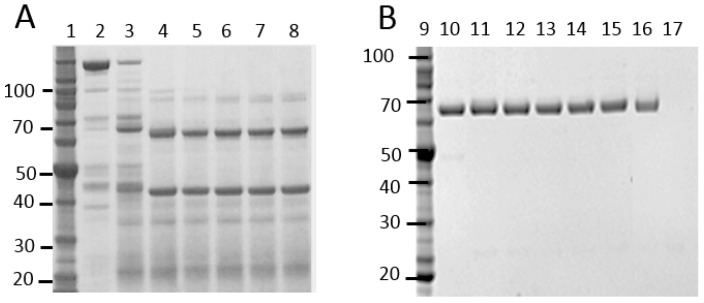
Digestion of MBP-IP3-7 (Panel **A**) and MBP-free, trypsin-digested, Superdex-purified IP3-7 (Panel **B**) with WCRW midgut digestive fluid. Approx. 0.5 mg/mL of IP3-7 proteins were exposed to the gut fluid, and the digestion was observed by SDS-PAGE. In Panel **A**, Lane 2 was MBP-IP3-7 not exposed to the gut fluid. Lanes 3–8 were MBP-IP3-7 digested with the gut fluid for 0 min (Lane 3), 15 min (Lane 4), 30 min (Lane 5), 1 h (Lane 6), 2 h (Lane 7) and 4 h (Lane 8). In Panel **B**, Lane 10 was MBP-free IP3-7 not exposed to the gut fluid. Lanes 11–16 were MBP-free IP3-7 incubated with the gut fluid for 0 min (Lane 11), 15 min (Lane 12), 30 min (Lane 13), 1 h (Lane 14), 3 h (Lane 15) and 16 h (Lane 16). Lane 17 was the gut fluid only. Lanes 1 and 9 were BenchMark™ Protein Ladder (Life Technologies, Carlsbad, CA, USA) with approx. molecular weights in kDa.

Digestions of MBP-IP3-7 in WCRW gut fluid were observed by SDS-PAGE (Figure 6). Within 15 min, the MBP-IP3-7 fusion was digested down to two 65 kDa and 44 kDa polypeptides. The 44 kDa polypeptide was considered to be MBP because of the size. At 0 min, MBP-IP3-7 showed two distinct bands (Figure 6, Lane 3) slightly larger than the 65 kDa and 44 kDa bands which were not seen at and after 15 min and after that. This observation suggests that not only the junction between MBP and IP3-7 but also a site between the leader sequence and the mature toxin, or even within the leader sequence, were possible sites for WCRW gut proteases. After IP3-7 was digested down to 65 kDa, it was resistant to any further digestion. The absence of the 55 kDa polypeptide indicates that the loop between α3 and α4 of IP3-7 was resistant to WCRW gut proteases. The MBP-free, trypsin-digested 65-kDa IP3-7 was purified by Superdex 200 column chromatography as shown in Figure 4 and exposed to the gut fluid. The 65-kDa toxin was completely resistant to the insect proteases (Figure 6, Panel B).

## 3. Discussion

A new Cry protein, Cry8Hb, was found in a Bt strain active against WCRW. Its primary sequence is similar to that of Cry8Ha. The host Bt strain of Cry8Ha, Bt185, was reported to be active against Asian cockchafer, *Holotrichia parallela* [16]. When two primary peptide sequences were compared between Cry8Ha and Cry8Hb, there were significant differences in Domain II. The 2D-structure (α-helices and β-strands) assignment made on Cry8Hb indicates that those significant differences are in β5-β9 make the Cry8Hb Domain II Loop 2 that connects β6 to β7 unique. The specific sequence difference suggests that Loop 2 of Cry8Hb is important for its WCRW specificity.

Yamamoto [17] reported that the Cry1A-type protoxin is highly soluble at a neutral pH. Upon ingestion, the Cry1A-type protoxin is activated by proteases in the insect digestive system. Trypsin can simulate the activation in vitro. When the Cry1A protoxin is digested with trypsin at pH8, the activated toxin precipitates. This precipitated toxin can then be solubilized in low or high pH away from pH7 indicating that its pI is around pH7. A similar observation was made with anti-WCRW-active Cry8Bb [6] and Cry8Hb (this study). During trypsin digestion of the Cry8Bb and Cry8Hb protoxins at pH8, those toxins precipitated. The gut fluid of Cry1A-susceptible insects is as alkaline as pH10 [18]. Under these conditions, the activated toxins, such as those of Cry1A and Cry8, remain soluble. Superdex gel filtration of trypsin-activated Cry3 and Cry8 toxins indicates that those toxins are monomeric in 25 mM CAPS-NaOH buffer, pH10. However, midgut fluid of WCRW is weakly acidic [19]. In acidic WCRW gut fluid, the activated toxin is likely to aggregate and precipitate as observed in vitro. On the other hand, the MBP fusion of the Cry8Hb toxin was highly soluble at pH8, and even at pH7, and its activity against WCRW was two-fold higher than that of the MBP-free toxin. Structural modeling of MBP-Cry8 fusion proteins indicated that the MBP molecule does not obstruct the receptor binding of Cry8 toxins, assuming the Domain II loops are involved in binding. It is possible that the MBP fusion binds to a receptor first, and then the MBP is excised from the Cry protein by gut proteases. We have found that the junction between MBP and the toxin is very sensitive not only to trypsin, but also to WCRW gut proteases in vitro. It is also possible that MBP is cleaved off by gut proteases before the toxin binds to a receptor. Regardless whether binding occurs before or after MBP excision, high solubility of the activated Cry protein in WCRW gut fluid appeared to be important for its activity. This solubility requirement was examined further. A synthetic *cry3* gene, IP3-1, was expressed in *E. coli* as a fusion with MBP and found that MBP enhanced the activity of IP3-1 13 fold.

Some technical issues of assaying insoluble proteins may exist. The activated Cry toxins are in 25 mM CAPS-NaOH buffer, pH10 to maintain the high solubility. On the other hand, the WCRW diet used in this study is about pH6. The pH of the diet-toxin mixture was pH6.1. At this low pH, the solubility of the toxin must not be maintained in the diet. The other possible issue is that high pH of the CAPS buffer may denature the Cry toxins. However, the toxin denaturation is unlikely since no significant activity reduction was observed with the activated toxin in the high pH CAPS buffer when compared with the protoxin activity in the neutral HEPES buffer.

Cry3 proteins are naturally truncated. The protein is not soluble in a neutral pH solution without a highly concentrated salt [12]. It has been reported that Cry3 proteins are not highly active against WCRW unless certain modifications are made to the proteins [2,3]. For example, Walters et al. [3] reported that engineering the loop between α-helices 3 and 4 increased the anti-WCRW activity. It seems the efficient digestion of the loop by chymotrypsin/cathepsin is important for the activity. A similar loop modification made on Cry8Bb enhanced the activity [10]. Our present study showed another way of enhancing the activity of WCRW-specific Cry proteins. During this study, we have found that MBP increases the solubility of those Cry8 proteins at a neutral pH and enhances the activity. Also, the activity enhancement with MBP was observed with a synthetic Cry3A-type protein called IP3-1.

DNA shuffling technology was applied to increase the anti-WCRW activity of the IP3 protein without MBP. The shuffling produced IP3 variants with both increased solubility at a neutral pH and high anti-WCRW activity. Superdex size-exclusion column chromatography indicates that the IP3 shuffled variant proteins are mostly monomeric at pH8. Those soluble and highly active variants share a common feature, namely, two mutations in the loop between α-helices 3 and 4 that make the loop resistant to WCRW gut proteases. Most mutations found in the shuffled IP3, particularly those making the protein acidic, are clustered on one side of the IP3 molecule on which MBP is likely to attach when fused to IP3. This assumption on the MBP-IP3 structure indicates that those mutations are likely to assume the solubility enhancing function of MBP.

DNA shuffling requires an efficient screening of a large number of variants. During this study, Cry8Hb was not chosen to conduct DNA shuffling to improve its solubility and activity because of its low activity even as an MBP fusion. The EC_50_ of MBP-Cry8Hb was 178 ppm. Without MBP, it was 390 ppm. To screen shuffled variants at this concentration, it requires 100 μL of approximately 3 mg/mL MBP-fusion protein samples. It is highly challenging to prepare a volume of protein samples at this concentration in a high throughput mode. In addition, there is no X-ray 3D structure for Cry8Hb available. Without the structure, it is difficult to understand the MBP-dependent activity enhancement by DNA shuffling as we have shown with Cry3. Furthermore, we found that the activity enhancement of Cry3 (IP3) with MBP is much higher than that of Cry8.

## 4. Materials and Method

### 4.1. High-Throughput Cry Protein Preparation from Bt Strains

A large number of Cry proteins from a Bt culture collection were screened for anti-WCRW activity. For screening, a Bt culture collection was arranged in seed culture plates made of 96-well microtiter plates containing LB-agar. Six production plates using Bel-Art™ Scienceware^®^ 96-Deep Well Plates (Scienceware, Wayne, NJ, USA) were prepared with 1 mL of a Bacillus sporulation medium [17] per well to produce spores and crystals. Those six production plates were inoculated with Bt cultures from one seed culture plate. The production plates were covered with AirPore tape (Qiagen, Valencia, CA, USA) and incubated in a shaker-incubator at 350 rpm, 30 °C for 72 h until the Bt cultures sporulated and produced insecticidal crystals. The spores and crystals from 6 production plates were collected in one combined plate as pellets by centrifuging at 3300× *g* for 30 min. Any soluble materials, particularly proteases, were removed from the spore-crystal pellets by suspending the pellet in each well in 2 mL 500 mM NaCl followed by centrifugation to re-collect spores and crystals. This process was repeated three times. The final spore and crystal preparations were suspended in 120 μL water per well and chilled on ice. To solubilize the crystals, 150 μL of pre-chilled 4% 2-mercaptoethanol, pH of which was adjusted to pH10.5 with 10 N NaOH, were added to the crystal suspension in each well, and the plates were centrifuged at 3300× *g* for 30 min. The supernatant was aspirated with a 96-channel pipette and desalted (buffer-exchanged) by Sephadex G25 gel filtration. Sephadex G25 was pre-saturated with 25 mM HEPES-NaOH buffer, pH8 to make the final Cry protein samples in this buffer, which is not toxic to WCRW even at 100 mM. The purity and concentrations of the Cry proteins in the eluate from Sephadex were determined by SDS-PAGE using 96-well E-PAGE™ 6% Protein Gels (Life Technologies, Carlsbad, CA, USA). The SDS-PAGE gel was stained with Coomassie Blue and photographed.

### 4.2. Bioassay of Cry Proteins

Insecticidal activity of purified Cry proteins was determined in 96-well microtiter plates as reported by Cong et al. [9] for lepidopteran insect species. In this study, neonate larvae of WCRW were used and scored in the same 0–3 numerical scoring system, which was based on larval growth and mortality. Since each Cry protein sample per dose was tested in 6 replicated wells, the theoretical maximum score was 18 at each treatment (a score of 3 assessed in all six wells). EC_50_ was defined as the toxin protein concentration that produced a total score of 9 (50% of the maximum score of 18). For the first-tier screening, only one appropriate dose was assayed to eliminate those with little or no activity. Active samples found by the first-tier screening were then assayed in a dose-response scheme to determine EC_50_. For some samples, the dose-response assay was repeated several times. The number of repetitions is reported in Results as “N” along with the 95% confidence limits or standard deviations.

### 4.3. Cloning and Sequencing a WCRW-Active cry Gene from DP7-F11 Bt Isolate

The WCRW-active *cry* gene was cloned from a genomic DNA preparation of the DP7-F11 Bt isolate. The genomic DNA sample was prepared from DP7-F11 cells harvested during the early logarithmic growth at A_600nm_ = 0.2 to 0.3 using QIAprep^®^ Spin Miniprep kit (Qiagen). Bt *cry* genes have been found on large plasmids. Since P2 in the QIAprep kit is an alkaline solution that may denature large plasmids, P2 was replaced with 1% SDS in water. A fragment of the WCRW-active *cry* gene in DP7-F11 was amplified from the genomic DNA preparation by 30-cycle PCR using Phusion^®^ High-Fidelity DNA Polymerases (New England BioLabs, Ipswich, MA, USA). The PCR was performed at 72 °C for 10 s per cycle using primers listed below for annealing at 37 °C for 30 s following the denaturation step at 94 °C for 10 s.

Forward primers (F); Reverse primers (R):

F1, 5′-GATAAAATCACTCAAATTCC; R-1, 5′-GGGATGAATTCGATTCGGTC0
F-2, 5′-GGCGAAATCACCCAAATACC; R-2, 5′-GGGATAAATTCGATTCGGTC
F-3, 5′-GATAAAATTACTCAGATTCC; R-3, 5′-GGAATAAATTCAATTCTATC
F-4, 5′-ACAAAAATCTCACAAATCCC; R-4, 5′-GGAATAAGTTCGAACTTATC
F-5, 5′-GATAAAATTACTCAAATTCC; R-5, 5′-GGGATGAATTCGATTTTGTC
F-6, 5′-AATAAAATCACTCAAATACC; R-6, 5′-GGGATGAGCTCGATTCGGTC
F-7, 5′-CAGAAAATCACCCAGATCCC; R-7, 5′-GGGATGAACTCGATACGATC
F-8, 5′-GAAAGAATCACACAATATCC; R-8, 5′-GGGATTAATTCCATTCTATC

These primers were based on the two conserved regions within the coding sequences for Domain III of Cry7 and Cry8 proteins [20]. All those primers were mixed and added to one PCR reaction mixture. DNA amplified by PCR was cloned in *E. coli* by using Zero Blunt^®^ TOPO^®^ PCR Cloning Kit for Sequencing (Life Technologies, Carlsbad, CA, USA). A large number of clones were sequenced, and the sequence analysis revealed one clone, which appeared to be a fragment encoding Domain III of a novel Cry protein. This gene was tentatively named RX002.

The 5′-flanking region of the PCR-amplified portion of RX002 including the sequence encodes Domain I and Domain II was amplified using APAgene™ genome walking Kit (Bio S&T, Montreal, QC, Canada) following the manufacturer’s recommended procedure. For this gene walking, four reverse primers were designed based on the partly determined sequence of the new RX002 gene as follows:

RX002-Ra, 5′-TATTAAGGGGCCAACTGCTCCGGCTAATGG
RX002-Rb, 5′-GCTCCGGCTAATGGAACACCCAATAACC
RX002-Rc, 5′-GATCTCCAGTATTGTAACCATTTACTG
RX002-Rd, 5′-AATCAGTTGTTGGTTCATTCGC

DNA produced by the genome walking procedure was cloned in the same TOPO^®^-cloning kit and sequenced. The 3’ flanking region of the cloned RX002 Domain III was amplified by PCR using the following primer set:

Forward primer, 5′-TGCGGAGGAATTGATAGATGCGG
Reverse primer, 5′-TCTGWYTGATTYSCACCATCACG

The forward primer was from the sequence of RX002, which had been determined by the first cloning step. Since the RX002 sequence appeared to be a Cry8 type, the reverse primer was obtained from a highly homologous region of known *cry8* genes encoding the protoxin domain by aligning those sequences. The PCR amplification produced most of the protoxin coding region except for the 200 to 300 bp towards the C-terminus based on the size of the RX002 protein. In addition, no stop codon was found in the amplified 3′ sequence. The missing end was then obtained by inverted PCR on *Taq*I-digested and circularized template DNA prepared from the DP7-F11 genomic DNA preparation using two primers designed from the RX002 sequence as follows:

Forward primer, 5′-GGAGATGGCTACGTAACGATTCG
Reverse primer, 5′-CCTTCTTTTCTTGCGGTAACACG

PCR amplified DNA was cloned into the TOPO cloning system as described above and sequenced. All fragments of RX002 were aligned to produce the full-length sequence. The RX002 gene sequence was confirmed by PCR amplification of the entire coding region in one reaction using primers designed from the assembled sequence and the DNA sample prepared from DP7-F11. The RX002 nucleotide sequence was submitted to the NCBI Genbank, and the peptide sequence was sent to the *Bacillus thuringiensis* Toxin Nomenclature Committee for new Cry protein assignment. The committee assigned a new Cry name as Cry8Hb1.

### 4.4. Cloning cry8Hb (RX002) in Bt and E. coli for Protein Expression

The *cry8Hb* (RX002) gene encoding the entire protoxin was cloned in a Bt-*E. coli* shuttle vector, pMAXY3206, to express it in a plasmid-cured, *cry*-minus Bt G8 strain. The cloning procedure and the pMAXY3206 shuttle vector constructed with pBluescriptKS(+) for *E. coli* and pBC16.1 for Bt were described by Cong et al. [9].

In addition to cloning the whole protoxin in Bt, a 5′ portion of the *cry8Hb* gene encoding a 75 kDa polypeptide (Met1- Lys699) was cloned in the pVER6805 vector to be expressed in *E. coli* as a fusion with MBP. The pVER6805 vector, which is similar to pMAL-pIII of New England BioLabs, has the same promoter and gene encoding MBP as those of the pMAL vector. In pVER6805, a poly histidine tag (6xHis) was attached to the MBP N-terminus for Ni-NTA affinity purification, and the *Eco*RI site was replaced with *Sph*I for cloning the newly found *cry* gene inframe after MBP between *Sph*I and *Bam*HI recognition sequences. The *Sph*I site utilizes the translation start ATG encoding Met. The toxin portion cloned in PVER6805 was amplified from the whole *cry8Hb* gene by PCR using the following two primers:

Forward primer, 5′-CAAAGCATGCGTCCAAATAATCAAAATGAATATG
Reverse primer, 5′-GTTTGGATCCTTACTTTGCCACCTCTAAATCGTTC

These primers add *SphI* and *Bam*HI recognition sequences (underlined) at the 5’ and 3’ ends of the *cry8Hb* gene when amplified by PCR. The forward primer sequence mutates the second amino acid residue of Cry8Hb from Ser to Arg. The PCR-amplified gene was digested with *Sph*I and *Bam*HI and cloned in pVER6805, which had been digested with the same enzymes. After cloning in Top10 *E. coli* (Life Technologies, Carlsbad, CA, USA), the DNA sequence of MBP-Cry8Hb was confirmed by Sanger DNA sequencing. All DNA sequencing during this study was done by MCLab (South San Francisco, CA, USA). Expression of the MBP-Cry8Hb fusion protein was performed in BL21 (DE3) *E. coli* (Life Technologies, Carlsbad, CA, USA).

### 4.5. IP3-1 Sequence Design and DNA Shuffling

A new Cry3-type protein termed IP3-1 was designed using Cry3Aa as the backbone on which mutations were introduced. Those mutations were chosen from sequence diversities among naturally occurring Cry3-type proteins available in the NCBI Protein Database. The IP3-1 gene was synthesized using *E. coli* codon usages by DNA2.0 (Newark, CA, USA). The synthesized IP3-1 gene was amplified by PCR using the following primers and cloned in pVER6805 as described above for *cry8Hb* (RX002).

Forward primer, CAAAGCATGCACCCTAACAACAGGTCAGAGC (underline: *Sph*I)
Reverse primer, GTTTGGATCCTCAGTTGACCGGGATGAACTC (underline: *Bam*HI)

When IP3-1 was cloned in pVER6805, the second residue was replaced with His by the forward primer to create the *Sph*I site. The nucleotide sequence of IP3-1 was disclosed in a patent [21].

DNA shuffling was performed on IP3-1 under mutational conditions as described by Stemmer [7,8]. The same primer set made for cloning the IP3-1 gene in pVER6805 was utilized to amplify shuffled DNA by PCR. The PCR-amplified DNA was digested with *Sph*I and *Bam*HI and cloned in pVER6805. *E. coli* BL21(DE3) was transformed with pVER6805 holding the shuffled genes and plated on LB-carb100 (carbenicillin 100 μg/mL) agar plates for picking individual clones.

About 5000 clones were selected in 96-well microtiter plates containing LB-carb100 agar for seed cultures. From each seed plate, 16 production plates consisting of 96-deep well Scienceware^®^ plates containing 1 mL MagicMedia™-carb100 (Life Technologies, Carlsbad, CA, USA) in each well, were inoculated. The plates were sealed with AirPore tape and incubated in a shaker-incubator running at 250 rpm at 37 °C for 8 h followed by 16 °C for 64 h. During the 16 °C incubation, the shuffled IP3 protein was accumulated in the BL21 cell as a soluble protein. The *E. coli* cells produced in one production plate were collected by centrifugation at 3300× *g* for 15 min. After supernatant was discarded, the cell suspension in the second plate was pipetted into the centrifuged plate, and the cells were packed on to the existing pellet by centrifugation. Repeating the process, all cells from 16 plates were combined in one plate. The centrifuged cells in each well were lysed in 400 μL of 2 mg/mL lysozyme solution in 50 mM sodium phosphate buffer, pH8 containing 5 mM MgCl_2_ and 0.25 unit/mL OmniCleave endonuclease (Epicenter, Madison, WI, USA) at 37 °C for 3 h followed by addition of 1% Triton X100. The lysed cells were centrifuged at 3300× *g* for 30 min to collect supernatant called clear lysate. The clear lysate preparations were screened for full-length IP3 expression by Western blotting using E-PAGE, iBlot^®^ nitrocellulose membranes (Life Technologies, Carlsbad, CA, USA) and anti-MBP antibody (Sigma-Aldrich cat# A3963, St Louis, MO, USA) following Life Technologies’ iBlot protocol. Full-length IP3 proteins confirmed by Western blotting were purified by Ni-NTA affinity chromatography in AcroPrep™ 1 mL 96-well filter plates with 1.0 μ glass fiber media (Pall Corporation, Port Washington, NY, USA). In the filter plate, 200 μL bed volume of Ni-NTA Superflow (Qiagen, ) was loaded per well, and approximately 400 μL of clear lysate was charged. The affinity purification was performed by following the manufacturer’s instruction except for centrifuging the filter plates stacked on 96-deep well receiver plates at 10× *g* for 3 min for sample loading, washing, and elution. Elution was made with 200 μL of 200 mM histidine in 50 mM sodium phosphate buffer, pH8 from each well. Histidine and sodium phosphate in the eluate were exchanged with 25 mM HEPES-NaOH buffer, pH8 by Sephadex G25 gel filtration in the AcroPrep™ filter plates. Sephadex G25 Superfine (GE Healthcare Life Sciences, Pittsburg, PA, USA) was suspended in 25 mM HEPES-NaOH buffer, pH8, and loaded to the filter plates to make a 700 μL bed volume in each filter well. The gel filtration was made in the Sephadex filter plate which works like a bundle of 96 spin columns.

The concentrations of Ni-NTA-purified proteins were determined by Caliper LabChip^®^ GXII capillary electrophoresis. The protein analysis was repeated at least three times until the final concentrations were considered reliable within the predetermined deviation of less than 10%. The concentration was normalized with 25 mM HEPES-NaOH buffer, pH8 using Hamilton Microlab Star before submission for bioassay.

### 4.6. Large-Scale Purification of Bt Cry Proteins

For purifying large amounts of Bt Cry proteins, especially alkaline-solubilized protoxins which are highly soluble (for example, 10 mg/mL) in Tris-HCl buffer at pH 8, the Bt cultures were grown in flasks, and the protoxin proteins were purified by size exclusion column chromatography as described by Yamamoto [17]. The purified Cry proteins were dialyzed in 25 mM HEPES-NaOH buffer, pH8 for insect bioassay. The dialyzed Cry proteins were analyzed by SDS-PAGE using NuPAGE^®^ Bis-Tris gels with MOPS buffer (Life Technologies, Carlsbad, CA, USA).

Selected variants of shuffled IP3 proteins cloned in pVER6805 as a fusion with 6XHis-MBP were purified from *E coli* cells produced in flasks. The proteins were liberated by disrupting the cells with 1% Triton X100 after the lysozyme treatment as described in Section 4.5. Ni-NTA Superflow (Qiagen) was used to conduct affinity purification in 1.5 × 12 cm BioRad Econo-Pac^®^ columns following Qiagen’s protocol. The eluate from Ni-NTA column was dialyzed in 25 mM HEPES-NaOH buffer, pH8. MBP was removed by digesting the fusion with trypsin at 1:50 enzyme to substrate ratio in 50 mM HEPES-NaOH buffer, pH8 at 37 °C for 1 h.

Trypsin-activated mature Cry toxins particularly those of Cry8Hb and IP3-1 were insoluble in the HEPES buffer at pH8. Almost immediately after trypsin was added to the MBP fusions of those Cry toxins for activation, the toxin proteins precipitated. Therefore, the trypsin-digested toxins were solubilized by raising the pH to 10.5 with 25 mM CAPS and NaOH and purified by Superdex 200 preparative column chromatography using 25 mM CAPS-NaOH buffer, pH10 as the elution-elution solvent. The column eluate was monitored by SDS-PAGE, and fractions contained the toxin were collected, combined and concentrated in Amicon Ultra-15, 10 kDa-cutoff, centrifugal filter units (Sigma-Aldrich, St Louis, MO, USA). The purified toxins in the CAPS buffer were subjected to the insect bioassay. The large-scale preparation of shuffled IP3 toxins after trypsin digestion was performed essentially in the same way as described above for IP3-1. For Superdex 200 column chromatography, 25 mM Tris-HCl buffer, pH8 was used as those variants remained soluble during and after the trypsin digestion. For the bioassay, Tris buffer was replaced with 25 mM HEPES-NaOH buffer, pH8. Concentrations of MBP-removed Cry toxins were determined by scanning the SDS-PAGE gel by Un-Scan-It-gel™ analysis software (Silk Scientific, Orem, UT, USA) using a known concentration of bovine serum albumin as a standard.

### 4.7. 3D Structural Analysis of IP3 Proteins

Several structural metrics of IP3-specific amino-acid residues were collected from a molecular property table of Cry3Aa generated by Discovery Studio software suite (Accelrys, San Diego, CA, USA). Hydrophobicity and pKa metrics of IP3-specific residues were based on those defined by Kyte and Doolittle [22] and by Creighton [23], respectively. The solvent accessibility subroutine calculated percent sidechain solvent accessibility with parameters of 240 grid points per atom and 1.4-Å probe radius. Space-filling protein structure models of Cry3Aa (1dlc) [12] and *E. coli* MBP (3hpi) [24] shown in Figure 5 were generated by RasMol Molecular Graphics [25].

### 4.8. Digestion of a Shuffled IP3 Protein in WCRW Midgut Fluid

Digestion of the shuffled IP3-7 protein in WCRW midgut fluid was observed in vitro. The gut fluid was obtained from gut samples dissected out from the third-instar WCRW larvae fed on germinating corn roots grown in the absence of soil under laboratory conditions. The dissected gut samples from 20 larvae were pooled in a single tube and centrifuged at 20,000× *g* at 4 °C for 20 min. The resulting supernatant was transferred to separate tubes in aliquots to use immediately or to be stored at −80 °C after they were flash-frozen in liquid nitrogen. The shuffled IP3 protein was diluted to 0.5 mg/mL in phosphate buffer saline containing 20% of the midgut fluid and incubated at 25 °C to observe the digestion in vitro. Two μL of this mixture were taken at predetermined time intervals and mixed with 20 μL of NuPAGE^®^ LDS sample buffer (Life Technologies, Carlsbad, CA, USA) that contained a reducing agent and Roche cOmplete™ Protease Inhibitor Cocktail obtained from Sigma-Aldrich (St Louis, MO, USA) at 2.5-fold higher concentration than the manufacturer’s suggested dilution. The samples mixed with LDS were heated immediately at 100 °C for 5 min and then analyzed by SDS-PAGE using NuPAGE^®^ 4-12% Bis-Tris precast gel (Life Technologies, Carlsbad, CA, USA).

## Figures and Tables

**Table 1:** Anti-WCRW activity of IP3 shuffled variants determined by dose-response insect assay.

Sample ID	EC50 (ppm)		
	Average	Std. Dev.	N
IP3-1	214	109	3
IP3-2	19	11.3	3
IP3-3	14.7	6.03	3
IP3-4	13.7	7.77	3
IP3-5	11.3	6.51	3
IP3-6	11.6	2.07	3
IP3-7	7.33	2.52	3
MBP-IP3-1	16.1	5.7	6

Std. Dev.: standard deviation, N: number of assay replications.

**Table 2A:** Sequence and structural analysis of IP3 variant proteins produced by DNA shuffling.

Cry3Aa1−							IP3-1			IP3-2			IP3-3		
Resd#	**AA**	2nd St	ɑ/β #	Sol Exp	Adj HyPo	pKa	**AA**	Adj HyPo	pKa	**AA**	Adj HyPo	pKa	**AA**	Adj HyPo	pKa
63	**K**	Helix	1	68%	−2.66	10.4	**K**	−2.66	10.4	**K**	−2.66	10.4	**K**	−2.66	10.4
97	**N**	Helix	2	97%	−3.39		**N**	−3.39		**N**	−3.39		**N**	−3.39	
106	**W**	Helix	2	1%	0		**L**	0.02		**L**	0.02		**L**	0.02	
117	**M**	Helix	2	2%	0.04		**I**	0.09		**I**	0.09		**I**	0.09	
119	**Q**	Coil		55%	−1.91		**Q**	−1.91		**Q**	−1.91		**Q**	−1.91	
140	**V**	Helix	3	1%	0.05		**F**	0.03		**F**	0.03		**F**	0.03	
152	**K**	Helix	3	97%	−3.77	10.4	**K**	−3.77	10	**E**	−3.87	4.3	**E**	−3.87	4.3
158	**R**	Coil		18%	−0.83	12	**R**	−0.83		**E**	−0.74		**E**	−0.74	
186	**I**	Coil		37%	1.66		**V**	1.03		**V**	1.03		**V**	1.03	
206	**F**	Helix	5	2%	0.05		**L**	0.06		**L**	0.06		**L**	0.06	
221	**E**	Coil		86%	−3.01	4.3	**E**	−3.01	4.3	**E**	−3.01	4.3	**S**	−0.86	
222	**K**	Helix	6	96%	−3.75	10.4	**K**	−3.75	10.4	**K**	−3.75	10.4	**S**	−0.96	
230	**K**	Helix	6	98%	−3.81	10.4	**H**	−3.13	6	**H**	−3.13	6	**H**	−3.13	6
232	**Q**	Helix	6	5%	−0.16		**Q**	−0.16		**Q**	−0.16		**Q**	−0.16	
258	**S**	Coil		54%	−0.43		**T**	−0.38		**T**	−0.38		**T**	−0.38	
292	**P**	Coil		48%	−0.76		**S**	−0.48		**S**	−0.48		**S**	−0.48	
294	**E**	Coil		55%	−1.91	4.3	**G**	0		**G**	0		**G**	0	
340	**I**	Sheet	2	0%	0		**I**	0		**V**	0		**V**	0	
346	**F**	Sheet	2	35%	0.97		**L**	1.32		**L**	1.32		**L**	1.32	
384	**K**	Coil		70%	−2.75	10.4	**K**	−2.75	7.3	**E**	−2.82	4.3	**E**	−2.82	4.3
468	**G**	Helix		0%	0		**A**	0		**A**	0		**A**	0	
472	**Q**	Sheet	10	14%	−0.49		**Q**	−0.49		**L**	0.55		**L**	0.55	
491	**L**	Sheet	11	4%	0.13		**F**	0.1		**F**	0.1		**F**	0.1	
496	**K**	Turn		24%	−0.95	10.4	**K**	−0.95	10.4	**K**	−0.95	10.4	**K**	−0.95	10.4
503	**M**	Sheet	12	52%	0.99		**T**	−0.37		**T**	−0.37		**T**	−0.37	
531	**R**	Coil		43%	−1.92	12	**G**	0		**G**	0		**G**	0	
557	**Y**	Coil		70%	−0.91	10	**Y**	−0.91	10	**Y**	−0.91	10	**Y**	−0.91	10
584	**F**	Sheet	19	18%	0.5		**F**	0.5		**F**	0.5		**F**	0.5	
589	**F**	Sheet	19	1%	0.04		**F**	0.04		**L**	0.05		**L**	0.05	
593	**I**	Coil		6%	0.26		**M**	0.16		**M**	0.16		**M**	0.16	
610	**S**	Coil		55%	−0.44		**S**	−0.44		**S**	−0.44		**S**	−0.44	
Average HyPo & pKa=					−0.94	9.55	****	−0.84	8.61	****	−0.81	7.51	****	−0.65	7.57
**HyPo, pKa reduction=**							****	**−0.1**	**0.94**	****	**−0.13**	**2.03**	****	**−0.29**	**1.98**

Resd# = residue number; AA = amino acid; 2nd St = secondary structure; ɑ/β # = ɑ helix. and β strand numbers used in the Cry3Aa X-ray structure; Sol Exp = side residue solvent exposure; HyPo = hydrophobicity index; Adj = adjusted by residue solvent exposure; Gray shade = mutations specific to shuffled variants.

**Table 2B:** Sequence and structural analysis of IP3 variant proteins produced by DNA shuffling.

	IP3−4			IP3-5			IP3-6			IP3-7		
Resd#	**AA**	Adj HyPo	pKa	**AA**	Adj HyPo	pKa	**AA**	Adj HyPo	pKa	**AA**	Adj HyPo	pKa
63	**R**	−3.07	12	**R**	−3.07	12	**E**	−2.73	4.3	**E**	−2.73	4.3
97	**D**	−3.39	3.9	**D**	−3.39	3.9	**N**	−3.39		**N**	−3.39	
106	**L**	0.02		**L**	0.02		**L**	0.02		**L**	0.02	
117	**I**	0.09		**I**	0.09		**I**	0.09		**I**	0.09	
119	**H**	−1.74	6	**H**	−1.74	6	**Q**	−1.91		**Q**	−1.91	
140	**F**	0.03		**F**	0.03		**F**	0.03		**F**	0.03	
152	**E**	−3.87	4.3	**E**	−3.87	4.3	**E**	−3.87	4.3	**E**	−3.87	4.3
158	**E**	−0.74		**E**	−0.74		**E**	−0.74		**E**	−0.74	
186	**V**	1.03		**V**	1.03		**V**	1.03		**V**	1.03	
206	**L**	0.06		**L**	0.06		**L**	0.06		**L**	0.06	
221	**E**	−3.01	4.3	**S**	−0.86		**E**	−3.01	4.3	**S**	−0.86	
222	**K**	−3.75	10.4	**S**	−0.96		**K**	−3.75	10.4	**S**	−0.96	
230	**H**	−3.13	6	**H**	−3.13	6	**H**	−3.13	6	**H**	−3.13	6
232	**Q**	−0.16		**Q**	−0.16		**H**	−0.15	6	**H**	−0.15	6
258	**T**	−0.38		**T**	−0.38		**T**	−0.38		**T**	−0.38	
292	**S**	−0.48		**S**	−0.48		**S**	−0.48		**S**	−0.48	
294	**G**	0		**G**	0		**G**	0		**G**	0	
340	**I**	0		**I**	0		**I**	0		**I**	0	
346	**L**	1.32		**L**	1.32		**L**	1.32		**L**	1.32	
384	**K**	−2.75	10.4	**K**	−2.75	10.4	**K**	−2.75	10.4	**K**	−2.75	10.4
468	**A**	0		**A**	0		**A**	0		**A**	0	
472	**Q**	−0.49		**Q**	−0.49		**Q**	−0.49		**Q**	−0.49	
491	**F**	0.1		**F**	0.1		**F**	0.1		**F**	0.1	
496	**K**	−0.95	10.4	**K**	−0.95	10.4	**E**	−0.97	4.3	**E**	−0.97	4.3
503	**T**	−0.37		**T**	−0.37		**T**	−0.37		**T**	−0.37	
531	**G**	0		**G**	0		**G**	0		**G**	0	
557	**Y**	−0.91	10	**Y**	−0.91	10	**H**	−2.25	6	**H**	−2.25	6
584	**L**	0.7		**L**	0.7		**F**	0.5		**F**	0.5	
589	**F**	0.04		**F**	0.04		**F**	0.04		**F**	0.04	
593	**V**	0.16		**V**	0.16		**M**	0.16		**M**	0.16	
610	**S**	−0.44		**S**	−0.44		**T**	−0.39		**T**	−0.39	
Ave=		−0.84	7.77		−0.68	7.88	****	−0.88	6.25		−0.72	5.9
**Reduction=**	****	**−0.1**	**1.78**	****	**−0.26**	**1.67**	****	**−0.06**	**3.3**	****	**−0.22**	**3.65**

Resd# = residue number; AA = amino acid; 2nd St = secondary structure; ɑ/β # = ɑ helix.and β strand numbers used in the Cry3Aa X-ray structure; Sol Exp = side residue solvent exposure; HyPo = hydrophobicity index; Adj = adjusted by residue solvent exposure; Gray shade = mutations specific to shuffled variants.
